# Expanding the Vocabulary of Peptide Signals in *Streptococcus mutans*

**DOI:** 10.3389/fcimb.2019.00194

**Published:** 2019-06-06

**Authors:** Justin R. Kaspar, Alejandro R. Walker

**Affiliations:** Department of Oral Biology, University of Florida, Gainesville, FL, United States

**Keywords:** peptides, bacterial communication, cell-to-cell signaling, genetic competence, transformation, LC-MS/MS, RNA-Seq, Ribo-Seq

## Abstract

Streptococci, including the dental pathogen *Streptococcus mutans*, undergo cell-to-cell signaling that is mediated by small peptides to control critical physiological functions such as adaptation to the environment, control of subpopulation behaviors and regulation of virulence factors. One such model pathway is the regulation of genetic competence, controlled by the ComRS signaling system and the peptide XIP. However, recent research in the characterization of this pathway has uncovered novel operons and peptides that are intertwined into its regulation. These discoveries, such as cell lysis playing a critical role in XIP release and importance of bacterial self-sensing during the signaling process, have caused us to reevaluate previous paradigms and shift our views on the true purpose of these signaling systems. The finding of new peptides such as the ComRS inhibitor XrpA and the peptides of the RcrRPQ operon also suggests there may be more peptides hidden in the genomes of streptococci that could play critical roles in the physiology of these organisms. In this review, we summarize the recent findings in *S. mutans* regarding the integration of other circuits into the ComRS signaling pathway, the true mode of XIP export, and how the RcrRPQ operon controls competence activation. We also look at how new technologies can be used to re-annotate the genome to find new open reading frames that encode peptide signals. Together, this summary of research will allow us to reconsider how we perceive these systems to behave and lead us to expand our vocabulary of peptide signals within the genus *Streptococcus*.

## Introduction

The idea that bacterial self-produced extracellular mediators could influence phenotypic behaviors first originated in the 1960s when Dr. Alexander Tomasz published on an environmental factor that was both heat-labile and sensitive to peptidases and could synchronize a *Streptococcus* population into a transformable state (Tomasz, 1965). At that time, it was the traditional view that bacterial populations were lacking mechanisms for specific intercellular communication and only cells of “higher organisms” coded for means to induce or reinforce the expression of many physiological properties. Coupled with the findings in *Vibrio* that bioluminescence required a self-produced factor in a term referred to at that time as autoinduction (Nealson et al., 1970), paradigms began to shift and it was recognized that bacterial populations could behave as a biological unit with considerable, although temporary, coordination among its members. Since that time, the study of bacterial communication or quorum sensing as it is now termed has rapidly expanded (Fuqua et al., 1994; Whiteley et al., 2017). We now know that the factor that Tomasz described is a small peptide or “pheromone” (Havarstein et al., 1997) and the phenomenon is widespread in Gram-positive controlling not only transformation, but also sporulation (Perego and Hoch, 1996) and production of extracellular toxins that impact virulence potential (Ji et al., 1995).

Today, the study of genetic competence (the transient phenotypic state leading to transformation as Tomasz described) remains a model pathway to dissect and explore cell-to-cell communication in Gram-positive bacteria. This is especially true within the genus *Streptococcus*, where extensive research progress has identified two distinct competence-activating signaling systems that divides the phylogenetic groups within the genus (Håvarstein, 2010). The well-studied ComCDE system reigns supreme in competence regulation among the Mitis and Anginosus groups that includes the popular model organism *Streptococcus pneumoniae*. ComCDE consists of a traditional two-component system signal transduction cascade composed of a histidine kinase and response regulator and is a model form of extracellular peptide signaling. The Pyogenic, Salivarius, and Bovis groups contain an intracellular signaling system, termed ComRS, comprised of a cytosolic transcriptional regulator that recognizes specific DNA sequences within promoter regions upon binding of its cognate peptide that is imported into the cells to regulate their competence activation. Finally there is the Mutans group of *Streptococcus* that contain both systems and regulate genetic competence dependent on the environmental conditions (Son et al., 2012). Being the oddball, *Streptococcus mutans* and its genetic competence circuit has served as an attractive model to sort out the interplay between the extracellular and intracellular signaling systems. This review will focus solely on the research and findings within *S. mutans*; for broader reviews on genetic competence within streptococci as a whole, readers are encouraged to view excellent reviews elsewhere that cover this topic (Jimenez and Federle, 2014; Johnston et al., 2014; Fontaine et al., 2015; Straume et al., 2015; Lin et al., 2016; Shanker and Federle, 2017).

**Figure 1:**
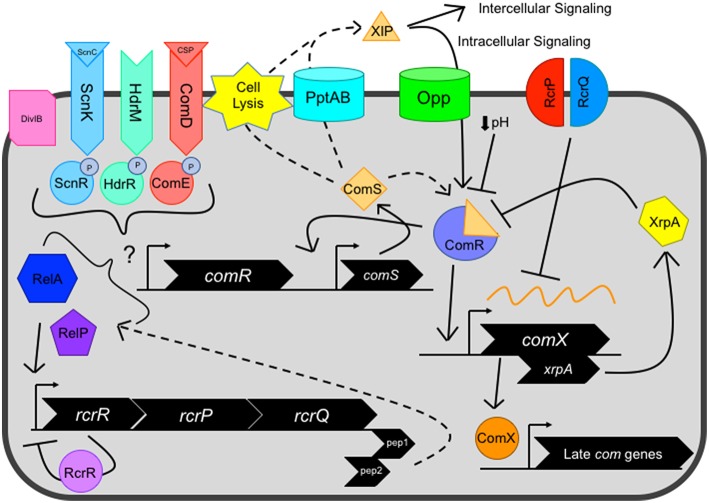
Current view of the genetic competence pathway in *S. mutans*. Competence activation is centrally mediated by the ComRS system, consisting of the Rgg-like transcriptional regulator ComR, and ComS, a 17-aa precursor of the 7-aa XIP peptide. ComS is processed into XIP and exported by unknown mechanism(s), potentially facilitated by dedicated transport systems such as PptAB or through cell lysis. Externalized XIP can activate nearby cells in an intercellular signaling mechanism or is re-imported by oligopeptide permeases (Opp) in an intracellular signaling manner. Cytosolic ComS or XIP interacts with the transcriptional regulator ComR, forming the ComR-XIP complex. The complex recognizes a select palindromic sequence termed the ComR-box, located within the promoter of both *comX* and *comS*, the latter creating a positive feedback loop for the system. Several negative regulators can also impact ComRS signaling, including decreased environmental pH and XrpA, located intragenically within the *comX* coding sequence on a separate reading frame that inhibits ComRS signaling through direct interaction with ComR. After competence activation, the alternative sigma factor ComX directs the RNA polymerase to a regulon consisting of late competence genes whose products make up the machinery needed for DNA uptake and potential homologous recombination of the single stranded DNA into the genome. However, the RcrRPQ operon, that is negatively regulated by RcrR, can shut down ComX production through overexpression of the ABC transporters RcrP and RcrQ that results in the processing/degradation of *comX* mRNA. Additionally, two peptides located at the 3′ end of the *rcrQ* coding region termed Pep1 and Pep2 are believed to modulate competence signaling through interactions with the (p)ppGpp enzymes RelA and RelP. Several distal regulators, including two component systems ComDE, ScnKR, and HdrMR, membrane protein DivIB and (p)ppGpp concentration mediated by the dual synthetase/hydrolase enzyme RelA can impact ComRS signaling.

Natural transformation in *S. mutans* was first described by Perry and Kuramitsu (1981). While not all recovered *S. mutans* isolates are transformable due to mutations in critical competence genes (Cornejo et al., 2013; Palmer et al., 2013), *S. mutans* has gained favorability for use as a model organism due to its ease of genetic manipulation and low biosafety level required for working with the organism (Lemos et al., 2013). The strain UA159 (early designations referred to this isolate as UAB577), was isolated in 1982 by P.W. Caufield at the University of Alabama Birmingham from a child with active cavities and was selected over the years for study due to its high degree of transformability over other *S. mutans* isolates (Murchison et al., 1986; Tao et al., 1993). Therefore, the wild-type and lab-adapted *S. mutans* has been selected for its peptide signaling and high degree of natural competence. In the last decade alone, numerous studies have enlightened our understanding of mechanisms by which these systems function as well as how these systems are in-tune with environmental sensing and stress response on a global scale to both habitat and foe (Figure 1). Along with these benchmark studies, more questions than answers have been raised with discoveries of novel peptides that appear to be associated with genetic competence leading to the mudding of once clear pathways by which these regulators operate.

## Bridging ComCDE and ComRS—A Missed Connection

Early work on the genetic competence circuit within streptococci was completed in the Mitis and Anginosus groups, where a secreted peptide termed competence stimulating peptide (CSP) was found to activate the system in a density-dependent manner (Håvarstein et al., 1995; Havarstein et al., 1997). A similar peptide was identified in *S. mutans*, and addition of a synthetic version of the peptide (sCSP) both to planktonic and biofilm-grown cells greatly increased the number of *S. mutans* transformants when donor DNA was present (Li et al., 2001). For the next decade, the CSP peptide and ComDE signaling system was seen as the activator for genetic competence in *S. mutans*. The system follows the classical extracellular peptide signaling mechanism of other Gram-positive bacteria, in which a ribosomally produced precursor (in this example ComC), is processed during export by the ComAB secretion system (Hui and Morrison, 1991). The proteolytic cleavage site arises immediately after a double-glycine motif, commonly observed at the end of leader peptides for non-lantibiotic peptide bacteriocins and CSPs produced from other streptococci (Havarstein et al., 1997). This cleavage results in a mature peptide that is 21 residues long. An 18 amino acid (aa) version of CSP that lacks the three C-terminal amino acid residues was purified from *S. mutans* strain GS5 culture supernatants that had been pre-treated with the 21-aa version of CSP to identify the resulting antimicrobial peptides and lantibiotics that would be produced (Petersen et al., 2006). It was later realized that the 21-aa version of CSP undergoes a second processing step by the SepM extracellular membrane-associated protease where the final three amino acids are removed to generate the functional 18-aa form. The 21-aa version alone is non-functional and is not able to activate competence or bacteriocin production (Hossain and Biswas, 2012; Biswas et al., 2016). Several sequenced clinical isolates of *S. mutans* do not encode the final three C-terminal amino acids of *comC* and generate the functional 18-aa upon secretion (Palmer et al., 2013). Once the extracellular concentration of functional CSP reaches a critical threshold level, it is bound and activates the transmembrane histidine kinase ComD which autophosphorylates and subsequently transfers the phosphate group to ComE, a cytoplasmic response regulator (Kreth et al., 2007; Xie et al., 2010; Martin et al., 2013). Recent study into the structure-function relationship between CSP and ComD binding have shown that hydrophobic residues in the central region of the CSP 18-aa are critical for ComD receptor binding (Bikash et al., 2018), that the first few N-terminal residues are dispensable for signaling (Syvitski et al., 2007), and that two of the three extracellular loops of ComD are required for CSP recognition while the third plays little role (Dong et al., 2016). Early studies of CSP and the ComCDE operon suggested the presence of a second CSP receptor, as addition of CSP to *comCDE* mutant biofilms partially restored architecture similar to that of wild-type biofilms (Li et al., 2002).

Beginning in 2009, evidence for another peptide signaling system that regulated genetic competence in *Streptococcus* began to emerge (Fontaine et al., 2009; Gardan et al., 2009; Monnet et al., 2014). Using *S. mutans* as the model for this pathway (Mashburn-Warren et al., 2010), this second route for competence induction was found to include a cytosolic Rgg-like transcriptional regulator termed ComR and a 17-aa peptide precursor encoded immediately downstream in ComS. ComS is processed into an active 7-aa peptide derived from the C-terminus of ComS called XIP (comX Inducing Peptide). In other streptococci, the ComRS pathway follows the traditional intercellular signaling paradigm in that the ribosomally translated ComS is exported into the extracellular space and cleaved by a protease(s) to yield XIP (Mashburn-Warren et al., 2010; Shanker and Federle, 2017). After XIP is produced and released, it is transported back into the cell by the oligopeptide ABC transporter Opp (Mashburn-Warren et al., 2010; Son et al., 2012). The re-imported XIP can then be specifically bound by ComR to form a dimeric ComR-XIP complex that functions as a transcriptional activator for the promoters of *comX* and *comS* (Fontaine et al., 2013; Khan et al., 2016; Talagas et al., 2016). The ComR-XIP complex recognizes a ComR-box, consisting of a 20-bp palindromic motif, whose core GACA/TGTC inverted repeat is conserved across ComRS-containing streptococci (Fontaine et al., 2009; Mashburn-Warren et al., 2010). The activation of *comS* creates a positive feedback loop that amplifies ComS and possibly XIP production (Son et al., 2012; Fontaine et al., 2013). The model for XIP release and processing may differ in *S. mutans*. While three independent research groups have detected XIP in supernatant fluids of *S. mutans* (Desai et al., 2012; Khan et al., 2012; Wenderska et al., 2012), the mechanisms for secretion or processing have not been identified in this organism to date (Kaspar et al., 2017). Notably, in *Streptococcus thermophilus* (Gardan et al., 2013), the Eep protease processes ComS to XIP, but an equivalent function for proteases in *S. mutans* with characteristics similar to Eep has not been demonstrated (Khan et al., 2012). Chang and Federle (Chang and Federle, 2016) have recently shown the ability of the ABC transporter PptAB to export small hydrophobic peptides used in Rgg-signaling systems in *Firmicutes*, however removal of *pptAB* in *S. mutans* only partially disrupted XIP signaling suggesting still other XIP export mechanisms are present.

The Mutans group of *Streptococcus* is unique in that most strains encode both ComCDE and ComRS pathways (Håvarstein, 2010). Differing environmental conditions, which include pH, redox and growth phase, influence the activity of each pathway (Hagen and Son, 2017). Addition of sCSP to growing cultures of *S. mutans* in a peptide-rich medium, such as BHI, results in activation within the entire cell population of transcription for the biogenesis of bacteriocins via direct binding of phosphorylated ComE to a conserved sequence in the promoter regions of these genes and operons (Kreth et al., 2007; Lemme et al., 2011). Transcription of *comX* can also be induced by CSP, but this generally occurs in only a subset of organisms in a population (Son et al., 2012). It does not involve direct binding of ComE to the *comX* promoter, and the underlying mechanism for CSP-dependent activation of *comX* is not well-understood (Kreth et al., 2007; Hung et al., 2011). Consistent with this observation, the ComCDE system of *S. mutans* appears to have evolved from a common ancestor of the BlpCHR system in *S. pneumoniae*, which does not regulate competence but does induce bacteriocins in the pneumococcus and some related organisms (Johnston et al., 2014; Shanker et al., 2016; Mignolet et al., 2018). For this reason, the ComCDE system has begun being referred to as BlpCHR in *S. mutans* or MutCDE (Reck et al., 2015; Shanker and Federle, 2017). The proximal regulator for direct activation of competence is the ComRS signaling system as removal of *comR* results in a non-transformable strain (Mashburn-Warren et al., 2010).

Several intriguing hypotheses have been put forth to connect the ComCDE and ComRS pathways and to explain how activation of ComCDE may result in *comX* upregulation. An early hypothesis suggested that a ComE-regulated bacteriocin, CipB, may have a moonlighting function in regulating ComRS expression as *cipB* mutants showed decreased transformation efficiency (Dufour et al., 2011). Further adding complexity to the system are the ScnCRK and HdrRM systems whose genetic alterations seem to impact ComR production and/or stability leading to decreases in *comX* expression (Okinaga et al., 2010b; Kim et al., 2013). ScnRK comprise a two-component system and 61-aa peptide in ScnC. Unlike other two component systems, ScnC is believed to block the activity of ScnRK when bound rather than activate it (Kim et al., 2013). HdrRM consists of a membrane bound inhibitor protein (HdrM) that antagonizes the activity of an associated LytTR family transcription activator (HdrR) (Okinaga et al., 2010a). Competence has been shown to be strongly activated either when HdrR is overexpressed or when the HdrM membrane-bound protein is inactivated via mutation (Okinaga et al., 2010b). Finally, a recent genome-wide transposon screen to uncover novel regulators of genetic competence in *S. mutans* highlighted DivIB, a protein proposed to participate in the regulation of cell division (Shields et al., 2017). Loss of *divIB* resulted in decreased P*comX* activity when stimulated with sCSP, however P*comX* remained active when sXIP was supplied. Importantly, bacteriocin production by the Δ*divIB* strain appeared not to be altered, suggesting that the ComCDE pathway in this strain was still functional despite a lack of *comX* activation by CSP. These data highlight DivIB as focus for future research into crosstalk between ComCDE and ComRS systems in *S. mutans*.

While it is unclear how ComCDE may affect ComRS expression, a feedback loop from ComRS to ComCDE was recently uncovered by two independent studies (Reck et al., 2015; Son et al., 2015b). The ComRS system was found to be required for *comE* activation by either CSP or XIP through ComX production. A ComX binding site (termed a *cin*-box motif) was found within the promoter of *comE*. P*comE* was unresponsive to addition of XIP to cell cultures after disruption of the *cin*-box (Son et al., 2015b). A second finding in this study was that bacteriocin activation via ComE required a low threshold of CSP (only 100 nM) and activated the entire population (Son et al., 2015b). This is in contrast to *comX* activation by CSP, where a higher threshold is required and only activates a subpopulation (Son et al., 2012). This finding raises the question of how increasing CSP concentrations can provide greater stimulation of *comX* if the activity of *cipB* is already saturated. One potential explanation is that the higher CSP concentrations activate an additional, unidentified signaling pathway in parallel to *cipB* that further stimulates the ComRS system and *comX*. One thing is clear: ComCDE signaling is much less sensitive to certain environmental signals compared to ComRS signaling. Thus, the two signaling systems may collect different information about the environment for different purposes. Bacteriocin production relies on the simpler ComCDE system, depending more on a population density input (true quorum-sensing behavior), whereas competence development under ComRS is sensitive to fine changes in environmental signals such as media type, carbohydrate source (Moye et al., 2016), oxidative stress (De Furio et al., 2017) and pH levels (Guo et al., 2014; Son et al., 2015a). The bidirectional flow of information between the two systems may therefore allow regulation of additional behaviors in response to combinations of inputs.

## Into the Milieu—Are Peptide Signals Meant to be Shared?

The activation of genetic competence in *Streptococcus* spp. has traditionally been viewed as a quorum sensing-like process. Secreted oligopeptides of the ComCDE or ComRS systems are excreted into the extracellular milieu, where they accumulate until a critical threshold concentration is overcome that activates their respective systems, usually via a positive feedback loop. This process is seen as a community-wide behavior, such that bacterial subpopulations are able to coordinate a multicellular-type response. Perhaps the best example of this behavior is the colonization of *Vibrio fischeri* in the light organ of the squid *Euprymna scolopes*, where high cell density of *V. fischeri* leads to bioluminescence that is activated by a cell-to-cell signaling process (Waters and Bassler, 2005). In streptococci, the peptides that activate genetic competence have been shown to be potentially induced by environmental stressors such as antibiotics, oxidative stress and nutrient/carbohydrate sources (Claverys et al., 2006; Ahn et al., 2007; Perry et al., 2009; Håvarstein, 2010; Moye et al., 2016; De Furio et al., 2017). The activation of competence can then be seen as a response by the population to challenging conditions in order to increase genome plasticity and fitness for the survival of a few.

For *S. mutans*, it was not clear whether ComRS signaling would be active within biofilms due to strict environmental requirements including a growth medium devoid of peptides and a pH > 6.5 left (Son et al., 2012, 2015a; Guo et al., 2014). Two separate studies of *S. mutans* grown in biofilm populations indeed found that ComRS signaling could be active under these conditions. In the first, sXIP peptide was supplied to growing biofilms at different time points (Shields and Burne, 2016). As the biofilms matured (20–23 h), increasing concentrations of sXIP were required to induce measurable *comX* activation, and only a fraction of the cells responded to the signal. This was in contrast to early biofilms (5–7 h), where robust *comX* activity was measured and the majority of the population responded. In a second study, a co-culturing system was designed such that one *S. mutans* strain (a “sender”) would overproduce the XIP peptide precursor ComS, exporting large amounts of XIP to the extracellular space so that a receiver strain, which was unable to produce XIP (Δ*comS*), would internalize the XIP via the Opp transport system and a response could be measured (Kaspar et al., 2017). This study concluded that XIP had the potential to act as a diffusible signal and that the XIP released by a sender could activate a nearby responder in biofilm populations. However, as more exopolysaccharide material was produced due to an increase in sucrose concentrations, signaling dramatically decreased showing that the intercellular signaling would be limited in range. Furthermore, signaling could not be successfully observed when the sender/receiver co-culture was embedded in an agarose matrix that viewed single cell populations over a limited time. The inability of the system to activate broader cell populations leaves open the question the efficiency of XIP as a true intercellular signaling system.

The importance of bacterial “self-sensing” or intracellular signaling in an autocrine-like mechanism has gained renewed focus in the last several years. In *Bacillus subtilis*, a co-culture system similar to the sender/responder experiment discussed above showed that the peptide-secreting cells displayed a stronger response to the signal than non-secreting cells due to self-sensing (Bareia et al., 2018). A similar finding was recently made in *S. mutans*, in that a Δ*comS* strain activated *comX* to a lower degree in response to exogenously-provided XIP compared to a wild-type strain (Underhill et al., 2018). A *comS*-overproducing strain has also been shown to self-activate independent to changes in the rate of medium replacement in a microfluidic device, suggesting that external XIP accumulation played little role in this strain's ability to activate competence (Underhill et al., 2018). The basis that XIP serves as intercellular signal has always been built on previous paradigms, detection of XIP by LC-MS/MS in supernates of *S. mutans* cultures that were grown to high cell densities (Khan et al., 2012; Wenderska et al., 2012), and the fact that filtrates of *S. mutans* cultures grown to high density induced P*comX* activity in reporter strains (Desai et al., 2012). However, the substantial data against this paradigm includes (i) requirement for an intact *comS* gene in order for CSP to elicit any *comX* response in a peptide-rich medium (Son et al., 2012), (ii) the XIP importer Opp not being required for the bimodal P*comX* activation seen in peptide-rich medium (Son et al., 2012), and (iii) fluid replacement not altering the induction of *comX* in a strain that overexpresses *comS* (Underhill et al., 2018). These findings must force us to reconsider the importance of extracellular XIP accumulated in the milieu and whether genetic competence is truly a cell-to-cell signaling behavior. Several other recent findings in other organisms support this anti-social behavior in signaling molecules. In *Pseudomonas aeruginosa*, an aggregate model that recapitulates the biogeographical properties of infection in the cystic fibrosis lung found that cell-to-cell signaling only occurs within but not between aggregates or cell clusters that are spaced some distance apart (Darch et al., 2018). Autocrine-like signaling via the ComCDE pathway was observed in *S. pneumoniae* due to cell chaining from exposure to the antibiotics aztreonam and clavulanic acid that extended the length of the limited competence window (Domenech et al., 2018). Specifically for XIP, it is known in *Streptococcus thermophilus* that XIP is not excreted into the extracellular medium and may be limited to just the bacterial cell surface, favoring a self-sensing activation model (Gardan et al., 2013).

A final piece to the puzzle in determining if *S. mutans* XIP is a real extracellular signal is finding (or not finding) a dedicated XIP exporter. As previously mentioned, there have been several attempts to find a XIP exporter that went unsuccessful including deletion of Eep and PptAB (Khan et al., 2012; Chang and Federle, 2016). This leaves room to postulate that either *S. mutans* XIP relies on a suite of non-specific exporters in its delivery to the external milieu, or XIP is released by a mechanism different from that in other streptococci. One hypothesis is that *S. mutans* cell lysis is the dominant pathway for release of XIP. After competence activation in *S. mutans*, a subpopulation of cells will undergo autolysis (Perry et al., 2009; Wenderska et al., 2012). Autolysis is mediated in *S. mutans* through the activities of encoded cell wall hydrolases that are a part of the ComX regulon (Khan et al., 2016), and possibly by intracellular bacteriocins (Perry et al., 2009). Release of XIP to bacterial kin could also be a part of this process—release of both a source of genetic material (eDNA) and activating peptide at the same time is a logical approach so that DNA is available as cells become fully competent. Support for this new idea arises from failed competence activation of a reporter strain grown in supernatant of a *S. mutans* strain lacking the major autolysin AtlA (Kaspar et al., 2017). Why would *S. mutans* lack a dedicated peptide export system that seems common in other streptococci? It may come down to lifestyle. *S. mutans* is an obligate biofilm organism, where regulated cell death and lysis is critical for biofilm maturation and stability through release of extracellular matrix components such as eDNA (Liao et al., 2014). *S. mutans* may have simply evolved in such a way that a dedicated XIP exporter was dispensable, and autolysis of cells within the biofilm community was sufficient. The explanation of cell lysis being responsible for XIP release is not out of line with previous data where a high optical density is needed for XIP to be identified within cellular supernatants and competence autoactivation in reporter strains (Khan et al., 2012; Wenderska et al., 2012). ComRS signaling in *S. mutans* may just be more reliant on timing (i.e., specific points in biofilm maturation) rather than true cellular density commonly observed in other quorum sensing systems.

## The Curious Case Of The Rcrrpq Operon

**Figure 2:**
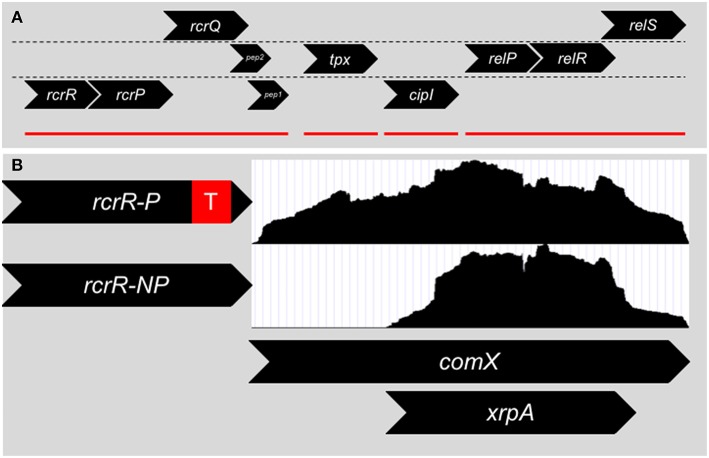
**(A)** Genomic organization surrounding the *rcrRPQ* operon of *S. mutans*. Genes are divided into their respective reading frames. *rcrR* encodes for a MarR-like transcriptional regulator that negatively regulates the transcription of the operon. *rcrP* and *rcrQ* are ABC-type multidrug/protein/lipid transporters. At the end of *rcrQ* are two small peptides encoded in separate reading frames. The start codon of Pep 1 overlaps with the stop codon of *rcrQ*. Pep 2's reading frame begins inside the *rcrQ* coding sequence and overlaps with the beginning of Pep 1. Downstream of the *rcrRPQ* operon is *tpx*, a thiol peroxidase, and *cipI*, a bacteriocin immunity protein. Finally *relP*, a small alarmone (p)ppGpp synthetase, along with the two component system *relRS* are further downstream. Mutations within *rcrRPQ* effect the expression of the *relPRS* operon and vice versa. Red lines denote operon structures within the region. **(B)** Two different mutants of *rcrR* are mainly used to investigate the operon that differ only by the presence of a transcriptional terminator (red box) within the antibiotic resistance cassette used to replace the gene. The Δ*rcrR*-P strain expresses the *rcrP* and *rcrQ* transporters at wild-type levels due to the presence of the strong transcriptional terminator and is hyper-transformable in terms of genetic competence, while the Δ*rcrR*-NP strain produces the transporters at 100x higher than wild-type background, leading to a non-transformable state. Differences in transformability between Δ*rcrR*-P and Δ*rcrR*-NP is the result of an altered *comX* transcript between the two strains. RNA-Seq read counts (white boxes) between the 5′ and 3′ ends of *comX* are even in the Δ*rcrR*-P background and a full transcript is produced. However, in the Δ*rcrR*-NP background, reads only map to the 3′ end and a smaller transcript is produced that corresponds to the intragenic coding region of *xrpA* that led to its discovery.

In 2011, Seaton et al. (2011) targeted and explored an operon in *S. mutans* that was linked to stress tolerance, (p)ppGpp accumulation and genetic competence and later termed RcrRPQ for Rel and Competence Related (Figure 2A). RcrR is a MarR-type negative transcriptional regulator that binds to the promoter region for the operon (Seaton et al., 2015). Downstream of *rcrR* are two ABC transporters, *rcrP* and *rcrQ*. Much of the research detailed by Seaton et al. discusses the phenotypes displayed by two separate mutants of *rcrR*: Δ*rcrR*-P contains a polar antibiotic resistant cassette in the replacement of the *rcrR* coding region (a strong transcriptional terminator is included after the antibiotic resistance gene), and Δ*rcrR*-NP contains a non-polar antibiotic resistant cassette (no transcriptional terminator is included). The ABC transporters *rcrP* and *rcrQ* in Δ*rcrR*-NP are upregulated 100-fold due to the loss of the negative regulator *rcrR* that controls the expression of the operon. Meanwhile, the presence of the transcriptional terminator greatly reduces the upregulation in Δ*rcrR*-P, resulting in the ABC transporters being expressed similarly to wild-type levels. Thus, we can assume that the phenotypes observed in Δ*rcrR*-P are from only the loss of *rcrR*, while those seen in Δ*rcrR*-NP may be due to both loss of *rcrR* as well as upregulation of *rcrP* and *rcrQ*.

Changes in the frequency of genetic transformation, both at basal levels as well as with addition of either *S. mutans* competence peptides, CSP or XIP, distinguish the Δ*rcrR*-P and Δ*rcrR*-NP mutants: Δ*rcrR*-P is hyper-transformable while Δ*rcrR*-NP cannot be transformed under any growth condition tested. This discrepancy between the two strains was made even more confusing by the early evidence that both *comS* and *comX* were upregulated in either strain (Seaton et al., 2011). It was clear that the competence phenotypes between the two strains could not be from defects in signaling, but rather from some type of post-activation inhibition. The answer was found when read accumulation between the two transcriptomes of either strain was compared using RNA-Seq (Kaspar et al., 2015). While it had appeared that *comX* was upregulated in Δ*rcrR*-NP via relative real-time PCR, only the 3′ half of the *comX* full-length transcript was present—the 5′ half was missing (Figure 2B). In fact, Δ*rcrR*-NP did not make a functional ComX protein at all (Kaspar et al., 2015). This could answer how Δ*rcrR*-NP was non-transformable, but the why is still left to ponder. Does RcrRPQ function as a kill switch for the competence circuit for *S. mutans*? One suggestion is that in a stressful condition that is sensed by the MarR-like regulator RcrR, the ABC transporters become upregulated, leading to the degradation of the 5′ *comX* mRNA transcript. Unfortunately, the signal for RcrR and the substrate for the transporters RcrP and RcrQ is unknown. The genomic location of the operon, however, may give us a clue. The (p)ppGpp synthetase *relP* and a two-component system termed *relRS* are encoded immediately downstream of RcrRPQ, with mutations of *rcrR* affecting *relP* transcription suggesting interplay between the two operons (Seaton et al., 2011). It has been shown that (p)ppGpp levels can affect competence activation through ComRS signaling, and subsequently deletion of the dual (p)ppGpp synthetase/hydrolase enzyme RelA can revert the non-transformable Δ*rcrR*-NP strain into a phenotype similar to its hyper-transformable counterpart Δ*rcrR*-P (Kaspar et al., 2016), further linking the *rcrRPQ* operon to (p)ppGpp. The operon could directly respond to (p)ppGpp accumulation or stressors that lead to changes in (p)ppGpp concentration within the cells.

The degradation of the 5′ *comX* transcript still left an abundance of the 3′ transcript remaining. This transcript does serve a purpose. Encoded within just a few bases of the transcript start was a 69 amino acid open reading frame (ORF). On the chance that this ORF coded for a functional protein, the start codon was mutated and evaluated for phenotypes (Kaspar et al., 2015). While the start codon mutant had minimal phenotype differences in an *S. mutans* wild-type background, the mutation completely restored the accumulation of full-length *comX* transcript as well as transformability in the Δ*rcrR*-NP strain. The ORF was named XrpA for *comX* Regulatory Peptide. Further research into the function of XrpA identified XrpA as a novel inhibitor of competence development through direct interaction with ComR, detailed through fluorescent polarization assays (Kaspar et al., 2018). While the exact mechanism by which XrpA binding to ComR inhibits ComRS signaling remains unknown, one hypothesis is that ComR is unable to form a dimeric complex when XrpA is bound (Talagas et al., 2016; Kaspar et al., 2018). XrpA does not appear to directly compete with XIP for the ComR peptide binding pocket (Kaspar et al., 2018). Several intriguing questions remain, including the translational mechanism by which the internal ORF XrpA is produced from the *comX* mRNA transcript, and whether XrpA is continuously active in the cell or is triggered by an environmental response that switches off ComRS signaling.

XrpA was not the only peptide that has been discovered that is connected to the RcrRPQ operon. Ahn et al. (2014) uncovered two peptides serendipitously at the end of the *rcrQ* ORF when two separately constructed *rcrQ* mutants, that differed only in the 3′ endpoint of the antibiotic cassette replacement, displayed altered phenotypes. Upon further review of the genome, two ORFs encoded on separate frames from *rcrQ* was noticed that could account for the phenotypic differences. One ORF's (*rcrQ* Pep1) start codon overlapped with the stop codon of *rcrQ*, while the other (*rcrQ* Pep2) overlaps with both the *rcrQ* ORF and the Pep1 ORF. When both peptides were removed in Δ*rcrR*-NP, the strain became hyper-transformable similar to what was observed when *xrpA* and *relA* was removed (Ahn et al., 2014; Kaspar et al., 2015, 2016). There was no change in the phenotypes of Δ*rcrR*-P, suggesting that de-repression of the operon is needed for the peptides to exert their effect. Individual deletion of either peptide through mutation of the start codon had marginal effects on growth rate in response to the competence activating peptide CSP as well as transformation efficiency—the best observable phenotypes were always when both peptides were deleted. Determining the function of the *rcrQ* peptides is currently ongoing. Interestingly, the activities of this operon appear limited to just *S. mutans*. Deletion of the operon in *Streptococcus gordonii* resulted in no changes to transformation efficiency but increased sensitivity to oxygen and reactive oxygen species in a similar manner to phenotypes observed in *S. mutans* (Shields and Burne, 2015). Perhaps further connecting the *rcrRPQ* operon with (p)ppGpp will shed new insights into the mechanisms by which the transporters and peptides integrate stress tolerance signals into the governance of competence regulation.

## More Peptide to be Discovered?

The ecological niche for the genus *Streptococcus* is narrow in scope, as almost all members are intimately associated and do not survive outside of a host, although some have been isolated in waste waters, on plants, and in milk and cheese (Mundt, 1982). Among them, the streptococci that inhabit the human oral cavity are uniquely adapted to the continuum of environmental insults as well as microbial antagonism within biofilm populations (Bowen et al., 2017). These challenges include rapid fluctuations in carbohydrate concentrations and other nutrient availability, exposure to environmental oxygen, and reactive oxygen species produced by competitors, reductions of environmental pH and exposure to the metabolic by-products of the other organisms that comprise oral biofilms (Lemos et al., 2013). To adapt to such constantly changing conditions, the microbiota employ numerous systems to optimize their gene expression patterns, cellular physiology, and fitness. For example, the *S. mutans* genome contains 14 two component systems and a single alternative sigma factor (*comX*) that together help to integrate sensory inputs from the environment or cell envelope into appropriate genetic and physiologic responses. Isolates of *S. mutans* have genomes that are about 2 megabases (Cornejo et al., 2013). This is comparatively small and in contrast to other host-associated and free-living organisms that have substantially larger genomes and a much greater repertoire of two component systems and alternative sigma factors to govern gene expression in response to specific environmental stimuli. Thus, *S. mutans* has evolved in a way that, instead of using specific sensing proteins, uses a set of global transcriptional regulators, conserved stress-response circuits (molecular chaperones, protease chaperones), monitoring of metabolic status (phosphosugar pools, HPr, acetyl phosphate levels), and sugar-specific permeases to interpret the environment and integrate this information with the physiologic status of the cells to fine-tune gene expression patterns and virulence capacities. Additionally, these bacteria must also coordinate mechanisms that allow for their establishment and growth among a consortia of other microbes in competitive interactions (Bowen et al., 2017). Therefore, it would not be surprising to find that these bacteria have encoded numerous small peptide/protein mediators to optimally monitor and adapt cell physiology to these numerous affronts to account for their small genome size.

**Figure 3:**
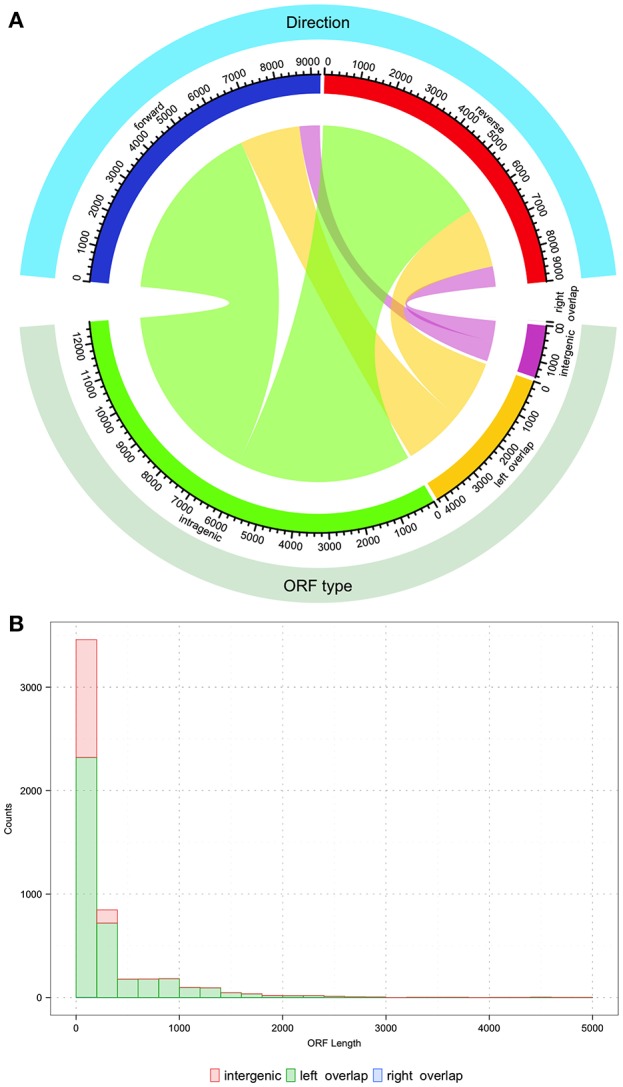
Description of potential coding open reading frames (ORFs) within the *S. mutans* UA159 genome. The *S. mutans* UA159 genome was downloaded from NCBI (Biosample SAMN02604090, Accession PRJNA333) with all annotations and loaded into Geneious (v 11.1.4). All ORFs (>100 nucleotides) were predicted with Geneious. **(A)** Chord plot showing the relationship between the type of predicted ORF and the direction (forward or reverse strand). From the total of 18,276 predicted ORFs, around 69% (12,599 ORFs) are completely intragenic or within another coding sequence. The count of ORFs that overlap with another on its 5′ end is 23% (4231 ORFs) and < 0.001% (1 ORF of length 123 bp) that overlap on its 3′ end. Finally, 8% (1445 ORFs) are located entirely within intergenic regions. Regarding the orientation of the ORFs, from the plot it can be concluded that roughly half of the total ORF count are oriented in opposite directions. Chord plot was generated with the function “chordDiagram” from the R package “circlize” (Gu et al., 2014). **(B)** Histogram of the nucleotide length distribution for intergenic, left, and right overlapping ORFs. Histogram was generated with the R package “ggplot2” (Wickham, 2016).

After uncovering the unusual *xrpA* and the *rcrQ* peptides, one must ask if we are underestimating the number of small peptides/proteins found within the genomes of streptococci that impact the fitness and virulence potential of these organisms. Simple analysis of the *S. mutans* UA159 genome shows that there are over 18,000 ORFs that have the potential to code for proteins >100 nucleotides in length. At the time of the *S. mutans* genome sequencing in 2002, 1,963 ORFs were assigned using the ORF prediction software GLIMMER 2.0 with the resulting annotations still in use today (Delcher et al., 1999; Ajdić et al., 2002). Through about two decades of research after the publication of the original genome, we know some of the resulting annotations are incorrect and ORFs containing peptide precursors such as ComS were not identified. This leaves open the real possibility that some protein/peptides encoded within small ORFs are overlooked that either serve as signaling molecules or act as small protein effectors. There are over 4,000 small ORFs that overlap with another ORF similar to the *rcrQ* peptides, 12,000+ ORFs that are intragenic to another larger ORF such as the relationship between *comX* and *xrpA*, and greater than a 1,000 ORFs that are intergenic that have no assigned coding function (Figure 3). These numbers highlight the potential that we may be still unaware of critical factors that shape bacterial metabolism, responses, and overall physiology.

Several advancements in technologies are notable toward the future pursuit of these unidentified factors. One popular technique for detection of low molecular weight proteins and peptides is liquid chromatography-tandem mass spectrometry (LS-MS/MS). The power of this technique was recently shown by a study of peptidic small molecules produced both in monocultures of oral bacteria isolates as well as in *in vitro* multispecies biofilms (Edlund et al., 2017). Only 2.2% of the peptides produced in these cultures could be putatively annotated, and the diversity of secreted peptides changed over growth of the biofilm cultures. This study reinforces the perception that we are severely lacking in our understanding of secreted factors, especially in the context of complex communities where cell-to-cell signaling, cooperation and antagonism interactions shape microbiome composition and persistence. While LS-MS/MS can determine the nature of small peptides present in a given sample, it's a greater challenge to resolve where in the genome these peptides originated. Terminomics, or the study of protein terminal sequences, is able to assist in determining additional non-annotated ORFs as well as ORF boundary adjustments from the original computational prediction (Dandekar et al., 2000; Jaffe et al., 2004; Hartmann and Armengaud, 2014; Berry et al., 2016). These strategies aim to identify the N-termini within an entire proteome through both N-termini enrichment strategies, such as *in vivo* acetylation (Chen et al., 2013), as well as bioinformatic data analysis (Hartmann and Armengaud, 2014). To date, proteogenomics has been used to re-evaluate the genomes of over 20 bacterial species, including relevant human pathogens *Helicobacter pylori* (Müller et al., 2013), *Mycobacterium tuberculosis* (Kelkar et al., 2011), *Salmonella* Typhimurium (Ansong et al., 2011), and *Yersinia pestis* (Payne et al., 2010). Specific selection of the N-termini is more beneficial toward the discovery of small peptides rather than traditional mass spectrometry as the technique reduces the high complexity and dynamic range of peptide fragments present in the sample, decreases the amount of peptides that may go undetected from poor ionization and those that lack charged residues, and eases biases present in traditional data analysis pipelines due to poor or low spectrum present from each peptide (Duncan et al., 2010; Berry et al., 2016).

The emergence of next-generation genomics and emerging transcriptomics technologies also offers opportunities to re-characterize a genome. These techniques are more user-friendly and do not come at a high cost burden compared to proteogenomics. Whole transcriptome shotgun sequencing, commonly referred to as RNA-Seq, allows for the annotation of transcriptional features at single-nucleotide precision in a strand-specific manner (Croucher and Thomson, 2010; Creecy and Conway, 2015). While RNA-Seq is commonly used in bacterial studies to determine differential expression of transcripts between strains and/or conditions, RNA-Seq can also be used to define operon architecture, identify promoters, transcriptional start sites, terminators, and antisense RNAs (Conway et al., 2014; Sharma and Vogel, 2014; Thomason et al., 2015). Proper identification and annotation of these features within a genome may reveal novel transcripts not previously recognized, such as the case with *S. mutans xrpA* (Kaspar et al., 2015). One pitfall of this analysis is pervasive transcription in non-canonical locations, leading to false interpretations about potential coding regions (Wade and Grainger, 2014).

RNA-Seq has also led to the development of derivative technologies that follow a similar protocol of RNA sequencing but with a specific focus rather than the entire transcriptome. One of these newer technologies is ribosomal profiling (Ribo-Seq) that maps the exact positions of ribosomes on transcripts by nuclease footprinting and subsequent RNA sequencing (Ingolia et al., 2009, 2012). Prior to cell harvest, a drug such as chloramphenicol is added to cultures to pause actively translating ribosomes. Purified mRNAs are then treated with nucleases to degrade regions that are not protected by the ribosome, leaving only 20–40 bp footprints that are mapped to the original genome or mRNA such that the location of a ribosome can be determined (Ingolia et al., 2011). Ribo-Seq takes transcriptome profiling a step further to determine which transcripts are actively being translated at the time of cell harvest. Ribo-Seq can be used to answer several key questions—it can be used as a quantitative proteomics tool to monitor known and new protein synthesis, measurement of protein synthesis rates which may be clues of posttranscriptional regulation, determination of ORF start codons and locations of translational pausing (Brar and Weissman, 2015). In the hunt for small proteins and peptides, the location of ribosome density along a given mRNA can be a critical sign that a specific ORF is actually translated, rather than being just pervasive translation. Two key studies in *Escherichia coli* have shown this proof of concept. In the first, ribosomes were paused with tetracycline that led to an accumulation of ribosomes at start codons (Nakahigashi et al., 2016). This technique was used to reannotate the N-termini of many known ORFs while also leading to the discovery of several unannotated ORFs in intergenic regions. In the second, ribosome elongation and initiation data was combined using both Onc112 and retapamulin which appear to be better alternatives than tetracycline for initiation site determination (Seefeldt et al., 2015; Meydan et al., 2019; Weaver et al., 2019). Predicted small proteins were then tagged, validating 38 of the 41 predicted (Weaver et al., 2019). Included in the validation were several proteins that contained substantial overlap with another ORF but encoded on a separate frame. While this technique offers several clear advantages for new ORF discovery and reannotation of the genome, users should be cognizant that this is still a relatively new technology that can be significantly impacted by the drug choice for stalling of ribosomes and the method chosen for rapid freezing of samples (Mohammad et al., 2019). Ribo-Seq protocols continue to evolve.

While there are several methods available for the discovery for new small peptides/proteins, the best approach in the future may actually be combining technologies. For instance, ribosome profiling may be first used for prediction of new start site initiations that is followed up by confirmation using proteogenomics and N-terminal mapping of peptide samples (Koch et al., 2014). While these high-throughput techniques offer speed and ability to re-map entire genomes at a time, validation of new peptides and proteins may still come down to tagging a protein of interest and visualizing a band on a gel. As these techniques become more popular and the bar for usage becomes lower in terms of cost and simplicity to the user, a mass re-evaluation of genomes to find hidden gems we may have missed could be on the horizon.

## Conclusion and Prospects

The study of cell-to-cell signaling in *Streptococcus* has provided a valuable model system to understand complex processes such as gene regulation, stress response, signal perception and transduction, and subpopulation and community behaviors. We are now approaching 25 years since the first characterization of CSP in *Streptococcus pneumoniae* (Håvarstein et al., 1995), yet we continue to make new discoveries that lead us to re-evaluate our understanding and realize the complexity by which these systems are controlled. This is especially true in *S. mutans*, where the findings of XrpA and inclusion of RcrRPQ into the story has only complicated matters. While there are many lingering questions, they should serve as examples to broaden our search for novel regulators that critically impact the physiology of key pathogens. We should be excited by the prospect that there may still be more out there currently hidden in the genomes of Gram-positive bacteria. One recent example is the finding of a leaderless secreted peptide consisting of only 8 amino acids in *Streptococcus pyogenes* that controls the production of the major virulence factor SpeB (Do et al., 2017). This determination of a critical molecular mechanism in a previously undescribed regulatory pathway offers hope for new therapeutic intervention that was previously not recognized. As we move closer to a post-antibiotic world and where manipulation of one's microbiome may prove to be a key curative strategy, these new research methods and findings should offer new approaches that move us closer to the selective targeting, disruption and removal of major pathogenic organisms.

## Author Contributions

JK contributed to conception, design, drafted, and critically revised the review. AW contributed to the figures, design, and critically revised the review. All authors gave final approval to the review and agree to be accountable for all aspects of the work.

### Conflict of Interest Statement

The authors declare that the research was conducted in the absence of any commercial or financial relationships that could be construed as a potential conflict of interest.
